# Inverse Source Data-Processing Strategies for Radio-Frequency Localization in Indoor Environments

**DOI:** 10.3390/s17112469

**Published:** 2017-10-27

**Authors:** Gianluca Gennarelli, Obada Al Khatib, Francesco Soldovieri

**Affiliations:** 1 Institute for Electromagnetic Sensing of the Environment, National Research Council of Italy, Via Diocleziano 328, Napoli 80124, Italy; soldovieri.f@irea.cnr.it; 2 Obada Al Khatib is with the Faculty of Engineering and Information Sciences, University of Wollongong in Dubai, Block 15, Dubai Knowledge Park, 20183 Dubai, UAE; obadaalkhatib@uowdubai.ac.ae

**Keywords:** data fusion, inverse source, RF localization

## Abstract

Indoor positioning of mobile devices plays a key role in many aspects of our daily life. These include real-time people tracking and monitoring, activity recognition, emergency detection, navigation, and numerous location based services. Despite many wireless technologies and data-processing algorithms have been developed in recent years, indoor positioning is still a problem subject of intensive research. This paper deals with the active radio-frequency (RF) source localization in indoor scenarios. The localization task is carried out at the physical layer thanks to receiving sensor arrays which are deployed on the border of the surveillance region to record the signal emitted by the source. The localization problem is formulated as an imaging one by taking advantage of the inverse source approach. Different measurement configurations and data-processing/fusion strategies are examined to investigate their effectiveness in terms of localization accuracy under both line-of-sight (LOS) and non-line of sight (NLOS) conditions. Numerical results based on full-wave synthetic data are reported to support the analysis.

## 1. Introduction

In recent years, the technological progress and widespread deployment of wireless devices have stimulated numerous research studies on radio-frequency (RF) localization in indoor environments. Indoor positioning attracts notable interest in many real-life applications and constitutes the core of several location-based services. For instance, indoor navigation [1,2] in large public facilities (airports, malls, etc.) is necessary as the conventional Global Positioning System (GPS) does not work owing to the attenuation of the GPS signal through building walls. Indoor navigation services are valuable for assisting visually impaired people or guiding tourists inside museums.

Accurate real-time people-tracking based on wireless technologies and sensor networks is required more than ever. Due to the ageing population in modern societies, there is a growing need to monitor elderly people while they are at home or in other buildings [3,4,5]. Automated location tracking of people and goods can also improve efficiency in logistics [6] and is helpful during search and rescue missions (e.g., firefighting) to assist operators in the field [7].

Indoor localization technologies can require a dedicated infrastructure such as Radio Frequency Identification (RFID), Ultra Wide Band (UWB), ZigBee, or infrared systems [8]. Alternatively, ubiquitous Wi-Fi, Bluetooth, or cellular network coverage ordinarily available in most public or private buildings can be conveniently exploited, thus avoiding the installation of new hardware and reducing costs.

In the frame of indoor RF localization and tracking, it is common to distinguish among cooperative (active) and non-cooperative (passive) targets [9]. In the former case, the targets are equipped with a device emitting an electromagnetic signal, which is recorded by receiving sensors deployed in the environment. In passive (device-free) localization, the sensors illuminate the target whose location is determined by the variations of the RF signal scattered by the target and recorded by the sensors.

When it comes to active localization [10], Received Signal Strength (RSS) data are conventionally processed according to path-loss models and distance-based methods [11] or a fingerprint approach [12]. Other popular localization algorithms combine geometrical techniques (trilateration, triangulation, etc.) with different types of information such as Time of Arrival (TOA) [13], Time Difference of Arrival (TDOA), Angle of Arrival (AOA), etc. An alternative way to address the localization problem is provided by array-processing methods such as beamforming [14] or inverse source approaches [9] whose goal is to estimate the source position from the reconstructed source current distribution. An overview of the aforementioned strategies for wireless indoor positioning is reported in [6,8,9,15] and references therein.

When the targets are non-cooperative, data processing approaches have been proposed based on dedicated hardware or ubiquitous wireless infrastructure [16,17,18,19]. Recent studies have been focused on tomographic techniques to image the region of interest and follow its spatial variations over time. In this area, it is worthwhile mentioning Radio Tomographic Imaging (RTI) [20], the approach combining an electromagnetic inverse source modeling and classification based on a support vector machine [21], and microwave tomography techniques based on both coherent and incoherent inverse scattering models [22,23,24,25,26,27,28,29,30].

This paper deals with the RF localization of active targets in indoor environments. As is well known, the problem is challenging and the subject of active research. Apart from hardware issues (time synchronization, limited bandwidth, etc.), the main challenge is related to the non-line of sight (NLOS) operation and multipath phenomena [31]. In these settings, ranging techniques based on direct path components lose accuracy because they overestimate the target range. Positioning accuracies not lower than 1m have been reported under NLOS and unknown multipath [15]. Accordingly, data-processing strategies aimed at mitigating multipath effects and enhancing localization accuracy are indispensable.

Given this framework, this paper proposes and compares different data-processing strategies for RF localization at the physical layer under conditions of a lack of prior knowledge of the environment. The localization task is formulated as a frequency domain inverse source problem where the electromagnetic signals emitted by the source are collected by sensor arrays deployed around the scene under investigation. The focus of this work is to assess and compare the performance of several localization strategies for the case of both narrow-band and UWB RF sources. Most notably, array data and image-fusion strategies are proposed to enhance the localization performance. Note that some image-fusion concepts considered in this work have been applied in previous studies [32,33]. However, those studies dealt with radar-imaging problems where an inverse electromagnetic scattering problem was faced. It is also stressed that the proposed localization strategies have the advantage of computational effectiveness and do not require any prior knowledge of the environment. Overall, to best of authors’ knowledge, they have not been analyzed and compared so far in the literature.

The paper is organized as follows. Section 2 states the problem and the formulation of the inverse source approach. The imaging performance of the inverse source approach is detailed in Section 3, where source localization strategies are also introduced. Numerical results based on synthetic data are presented in Section 4 and concluding remarks follow in Section 5.

## 2. Problem Statement and the Inverse Source Approach

**Figure 1:**
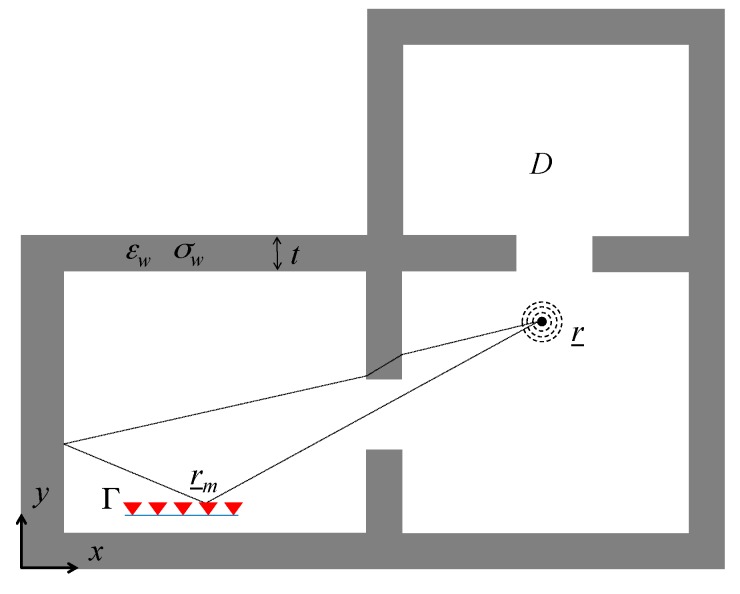
Geometry of the scenario. A radio-frequency (RF) source (black circle) radiates a wireless signal which is recorded by a sensor array (red triangles) deployed along the measurement line Г.

The two-dimensional (2D) indoor scenario represented in Figure 1 is considered. The investigated region D comprises adjacent rooms delimited by dielectric walls with relative dielectric permittivity $\varepsilon_{w}$, electric conductivity $\sigma_{w}$, and thickness *t*. A mobile RF source located at a generic point $\underline{r}=x\hat{x}+y\hat{y}\in D$ radiates a RF signal over the frequency interval $\Omega =\left[\omega_{min},\omega_{max}\right]$, with ω=2πf being the angular frequency. For sake of simplicity, the TM polarization with the electric field directed along the *z*-axis is considered. The electromagnetic signal radiated by the source propagates in the environment and is collected by a linear antenna array deployed along the measurement interval Γ.

Depending on the source and array location, each antenna in the array at ${\underline{r}}_{m}=x_{m}\hat{x}+y_{m}\hat{y}\in \Gamma$ may be in the line-of-sight (LOS) or NLOS region of the source. Specifically, the electric field recorded at each sensor location is given by the superposition of the direct path and multipath components (e.g., reflections, diffractions, etc.) in the LOS region while it is produced only by multipath contributions in the NLOS region. The exp(jωt) time dependence is assumed and suppressed.

According to [34], the measured electric field E is expressed by the radiation integral

(1) $$E\left({\underline{r}}_{m}\right)=\overset{}{\underset{D}{\iint }}G\left({\underline{r}}_{m},\underline{r}\right)J\left(\underline{r}\right)d\underline{r}=A\;J\;\;\;\;\;\;\;\;{\underline{r}}_{m}\in \Gamma$$

In the above formula, J is the unknown current distribution which is different from zero over the source region and zero elsewhere; G is the scalar 2D Green’s function of the scenario at hand; and $A:{ℒ}^{2}\left(D\right)\to {ℒ}^{2}\left(\Gamma \times \Omega \right)$ is a linear operator mapping the unknown space into data space. Such spaces are assumed to be Hilbert spaces of square integrable functions.

According to Equation (1), a linear inverse problem is established where the goal is to determine the unknown function J from the knowledge of the electric field E. Such an inverse source problem is ill-posed [35] and regularization schemes must be applied to achieve a stable solution.

Before addressing the inverse problem, it is necessary to evaluate the kernel of Equation (1), i.e., the Green’s function G. Owing to the complexity of the scenario, G is not available in closed form and it is necessary to resort to numerical methods such as, for instance, ray-tracing [36] or the finite difference time domain technique [37,38]. These methods require prior knowledge of the scene layout in terms of geometrical features (room shape, size, etc.) and electromagnetic wall properties (thickness, permittivity, conductivity). Moreover, in most practical cases, wall properties are not readily available and must be estimated from the data collected once the measurement system has been deployed. 

In this work, the focus is on the development of “blind” data-processing strategies for source localization which do not need prior knowledge of the environment or calibration data to estimate model parameters. Thus, when solving the inverse problem defined by Equation (1), the presence of the walls is neglected and RF signal propagation is assumed to occur in free space. Under this assumption, the Green’s function G is available in closed form [34]

(2) $$G\left({\underline{r}}_{m},\underline{r}\right)=\frac{j}{4}H_{0}^{\left(2\right)}\left(k_{0}R\right)$$

where $H_{0}^{\left(2\right)}\left(\cdot \right)\;$ is the zero-order Hankel’s function of the second kind’ $k_{0}=\omega /c$ is the propagation constant in free-space (c is the speed of light in vacuum); and $R=\left|{\underline{r}}_{m}-\underline{r}\right|=\;\sqrt{{\left(x-x_{m}\right)}^{2}+{\left(y-y_{m}\right)}^{2}}$ is the distance between the measurement position ${\underline{r}}_{m}$ and a generic point $\underline{r}$ in D.

It must be stressed that the adoption of the free space Green’s function instead of the inhomogeneous Green’s function introduces an approximation in the inverse source model that affects the localization accuracy in NLOS scenarios (e.g., see [38]). Nevertheless, this choice is justified by the need for simple and computationally efficient algorithms which do not require calibration data and are capable of operating in real-time. Moreover, as it will be shown, the positioning accuracy can be controlled and enhanced to a certain extent by properly selecting the most suitable measurement configuration and data-processing strategy [39,40].

In order to meet the efficiency requirement, the inverse problem in Equation (1) is solved by resorting to the adjoint operator $A^{†}$ of A [35], i.e.,

(3) $$\tilde{\;J}\left(\underline{r}\right)=A^{†}E=\iint^{\text{​}}G^{*}\left({\underline{r}}_{m},\underline{r}\right)E\left({\underline{r}}_{m}\right)d{\underline{r}}_{m}d\omega$$

where * denotes the conjugate operation.

The adjoint inversion defined by Equation (3) is well known in the frame of inverse problems [35] and is evaluated as the matrix product between the conjugate transpose of the matrix associated to the continuous operator 𝒜 and the data vector.

The spatial map defined by the amplitude of the reconstructed source current distribution $\tilde{\;J}$ normalized with respect to its maximum is referred to as tomographic image and denoted as ℐ.

## 3. Imaging Performance and Localization Strategies

### 3.1. Resolution Analysis

Before defining the localization strategies, it is worthwhile discussing the imaging performance achievable while solving the inverse source problem in order to gain a deeper insight into the problem. To this end, it is convenient to represent A in terms of its singular value decomposition (SVD) [35]

(4) $$A=\overset{\infty }{\underset{n=1}{\sum }}\sigma_{n}v_{n},\;\cdot u_{n}$$

where 〈 ,〉 denotes the scalar product; $\sigma_{n}$ is the set of singular values of A; and $v_{n}$ and $u_{n}$ are the singular functions, which provide orthonormal bases in the space of unknowns and data, respectively. The singular values $\sigma_{n}$ exhibit a slow varying behavior up to a critical index $N_{t}$, after which they decay exponentially [35,41,42].

By accounting for the SVD Equation (4), it follows that

(5) $$\tilde{\;J}=A^{†}E=\overset{\infty }{\underset{n=1}{\sum }}\sigma_{n}\langle E_{s},u_{n}\rangle v_{n}$$

It is seen from Equation (5) that the adjoint inversion scheme yields a stable solution since the singular values rapidly decay to zero beyond the critical index $N_{t}$. On the other hand, the faster variations of the source current distribution are lost with a consequent limitation on the spatial resolution [35]. The following analysis is based on the adjoint inversion scheme. A comparative study between linear inversion based on adjoint operator and truncated singular value decomposition (TSVD) is reported in [43].

A common figure of merit to foresee the achievable resolution is the point spread function (PSF), i.e., the regularized reconstruction of a point-like current. Upon considering a unitary impulsive current located at ${\underline{r}}_{0}$, i.e., $J\left(\underline{r}\right)=\delta \left(\underline{r}-{\underline{r}}_{0}\right),$ it turns out that the PSF has the following expression in terms of SVD:

(6) $$\mathrm{PSF}\left(\underline{r};{\underline{r}}_{0}\right)=\overset{\infty }{\underset{n=1}{\sum }}\sigma_{n}^{2}v_{n}\left(\underline{r}\right)v_{n}^{*}\left({\underline{r}}_{0}\right)\;$$

As is well known, the PSF peaks at the source position and exhibits side lobes whose amplitude progressively decreases away from the maximum [35]. This point suggests, as shown in next sub-section, a simple criterion for source localization.

An alternative way to evaluate the retrievable information on the unknown function is provided by the spatial spectral coverage, i.e., the set of retrievable spatial harmonics of the source current distribution. Unlike the PSF, the spectral content provides global information for current sources located anywhere in D rather than local information.

Consider Equation (6) and compute the Fourier transform of both sides with respect to the spatial variables x and y, i.e.,

(7) $$\mathrm{PSF}\left(\underline{k};{\underline{r}}_{0}\right)=\overset{\infty }{\underset{n=1}{\sum }}\sigma_{n}^{2}v_{n}^{*}\left({\underline{r}}_{0}\right){\hat{v}}_{n}\left(\underline{k}\right)\;$$

with $\underline{k}=k_{x}\;\hat{x}+k_{y}\;\hat{y}$ ($k_{x}$ and $k_{y}$ are the conjugated spectral variables corresponding to x and y), and

(8) $${\hat{v}}_{n}\left(\underline{k}\right)=\overset{}{\underset{D}{\iint }}v_{n}\left(\underline{r}\right)\exp\left(-j\underline{k}\cdot \underline{r}\right)d\underline{r}$$

Afterwards, the square amplitude of both sides of Equation (7) is computed, thus obtaining

(9) $${\left|\mathrm{PSF}\left(\underline{k};{\underline{r}}_{0}\right)\right|}^{2}=\overset{\infty }{\underset{n=1}{\sum }}\sigma_{n}^{2}v_{n}^{*}\left({\underline{r}}_{0}\right){\hat{v}}_{n}\left(\underline{k}\right)\;\overset{\infty }{\underset{m=1}{\sum }}\sigma_{m}^{2}v_{m}^{}\left({\underline{r}}_{0}\right){\hat{v}}_{m}^{*}\left(\underline{k}\right)\;$$

As the spectral functions involved in Equation (9) depend on the specific source position ${\underline{r}}_{0}$, the above formula is integrated over all possible values of ${\underline{r}}_{0}\in D$ in order to obtain global (average) spectral information. Consequently, we have

(10) $$\mathrm{SC}\left(\underline{k}\right)≜\overset{}{\underset{D}{\iint }}{\left|PSF\left(\underline{k},{\underline{r}}_{0}\right)\right|}^{2}d{\underline{r}}_{0}=\;\overset{\infty }{\underset{n=1}{\sum }}\;\overset{\infty }{\underset{m=1}{\sum }}\sigma_{n}^{2}\sigma_{m}^{2}\;{\hat{v}}_{n}\left(\underline{k}\right){\hat{v}}_{m}^{*}\left(\underline{k}\right)\overset{}{\underset{D}{\iint }}v_{n}^{*}\left({\underline{r}}_{0}\right)v_{m}^{}\left({\underline{r}}_{0}\right)d{\underline{r}}_{0}\;$$

Owing to the orthonormality of the singular functions $v_{n}$,

(11) $$\delta_{nm}=\overset{}{\underset{D}{\iint }}v_{n}^{*}\left({\underline{r}}_{0}\right)v_{m}^{}\left({\underline{r}}_{0}\right)d{\underline{r}}_{0}$$

with $\delta_{nm}$ being the Kronecker delta, it follows that

(12) $$\mathrm{SC}\left(\underline{k}\right)=\overset{\infty }{\underset{n=1}{\sum }}\sigma_{n}^{4}{\left|{\hat{v}}_{n}\left(\underline{k}\right)\right|}^{2}$$

We observe that the PSF and SC are intrinsic properties of the radiation operator A that are completely characterized once the measurement set-up and the inversion scheme have been established.

### 3.2. Localization Strategies

The RF source localization is performed starting from the knowledge of the tomographic image ℐ obtained after solving the inverse source problem. It is assumed that the current source is small in terms of radiating wavelength so that it can be treated as a point source. Thus, it is expected that the current distribution reconstructed via Equation (3) is essentially equivalent to a PSF, which reaches its maximum value at the true source location. Accordingly, the source position is estimated as the peak location of the reconstructed current distribution, i.e., 

(13) $$\underline{\tilde{r}}=\underset{\underline{r}\in D}{\mathrm{argmax}}\;ℐ\left(\underline{r}\right)\;$$

As will be shown, the localization accuracy achievable by applying the above criterion is highly dependent on the receiving array configuration. Therefore, it is interesting to investigate different array arrangements as well as deploying multiple receiving arrays in order to increase the spatial diversity, possibly enhancing the system’s performance.

According to the scenario previously shown in Figure 1, a horizontal (*x*-directed) antenna array is considered as the first sensing strategy of concern. In order to sense the scene from different measurement positions, it is also worthwhile examining the effect of the receiving array orientation. Therefore, the second sensing strategy analyzed here deals with a linear antenna array with its axis tilted with respect to the horizontal direction.

**Figure 2:**
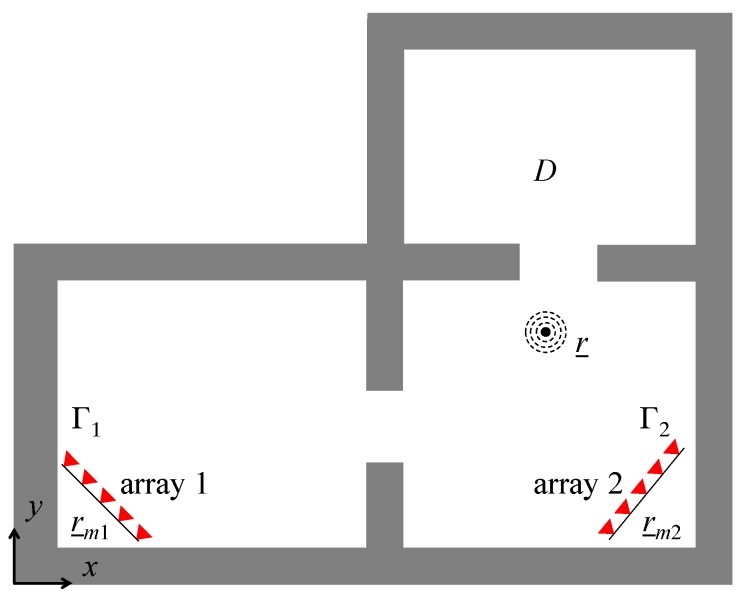
RF source localization with two receiving arrays. The arrays are located in different rooms and are tilted with respect to the *x*-axis.

Here, we investigate also cases where multiple receiving arrays are deployed in different rooms. The considered scenario illustrated in Figure 2 features two tilted arrays (array 1 and 2) located near the corners of two distinct rooms. The aim of such a sensing configuration is to increase the spatial diversity and improve the localization accuracy. However, to meet this goal, the information collected by the arrays must be “fused” to obtain a tomographic image suitable for localization. Two different fusion approaches are taken into account here.

The first approach deals with the fusion in the data domain. Thus, the radiation integral (1) at the basis of the inverse source approach reads as

(14) $$E\left({\underline{r}}_{m}\right)=\overset{}{\underset{D}{\iint }}G\left({\underline{r}}_{m},\underline{r}\right)J\left(\underline{r}\right)d\underline{r}=A_{U}\;J\;{\underline{r}}_{m}\in \Gamma_{1}\cup^{\text{​}}\Gamma_{2}$$

where $A_{U}:{ℒ}^{2}\left(D\right)\to {ℒ}^{2}\left(\Gamma_{1}\cup^{\text{​}}\Gamma_{2}\times \Omega \right)$ is the radiation operator mapping the space of unknown in the data space identified by the measurements of the two arrays. The model defined by Equation (14) is inverted by the adjoint operator to produce a tomographic image suitable for localization.

The second approach carries out the fusion in the image domain. In detail, two tomographic images ${ℐ}_{1}$ and ${ℐ}_{2}$ are obtained by inverting separately the datasets collected by arrays 1 and 2. Afterwards, ${ℐ}_{1}$ and ${ℐ}_{2}$ are combined to produce a composite image to be exploited for localization. Simple and cost-effective pixel-by-pixel additive and multiplicative image fusion are applied to produce the composite image [32,33].

The inverse source-based localization strategies that will be analyzed and compared in the next section are summarized and named with capital letters as follows:

- horizontal array (A)
- tilted array (B)
- tilted-array data fusion (C)
- additive-array image fusion (D)
- multiplicative-array image fusion (E)

## 4. Numerical Results

**Figure 3:**
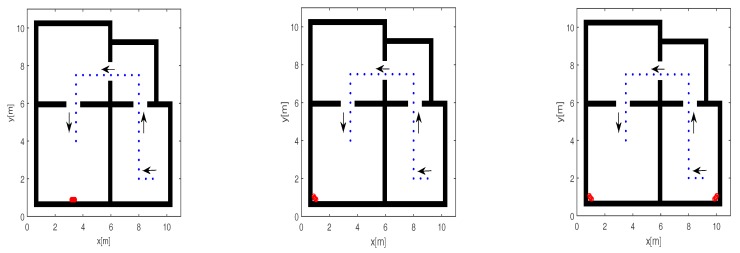
Geometry of the scenario and array configurations. The red circles indicate the receiving antennas and the blue points denote the positions of the RF source along its path. Horizontal array configuration (**left panel**). Tilted-array configuration (**middle panel**). Two-tilted arrays configuration (**right panel**).

This section presents the results of numerical tests carried out for the indoor environment displayed in Figure 3. The scene comprises four adjacent rooms delimited by homogeneous dielectric walls with a thickness of 0.3 m, relative permittivity of 4, and electric conductivity of 0.005 S/m. The RF source (blue points) moves within the rooms, starting from the bottom right room down to the room at the bottom left (see arrows). Thirty points with a uniform spacing of 0.5 m are considered to discretize the source path. The arrays (red circles) are 0.2 m long and composed by five isotropic antennas uniformly spaced at 0.05 m. The left panel of Figure 3 refers to the scenario where a single horizontal array is deployed close to one perimetral wall and located in the middle of the room along the *x*-axis. The middle panel of Figure 3 is concerned with the scenario where the receiving array is near the corner of the room and tilted to 135° with respect to the positive *x*-axis. The right panel of Figure 3 addresses the case where two tilted arrays with 135° and 45° tilt angles are deployed near the corners of two different rooms. The three sensing configurations depicted in Figure 3 enable the comparison of the inverse source data-processing strategies previously defined in Section 3.2.

The investigation domain *D* = [0.5, 10.5] × [0.5, 10.5] m^2^ is discretized into square image pixels having a side of 0.05 m. The RF source and receivers operate in the frequency band [2400, 2483.5] MHz. Such a narrow band allows simulating the operation of a Bluetooth device and addressing the indoor positioning with ubiquitous and low-cost hardware solutions. The case of an UWB RF source is also taken into account as a benchmark for comparison purposes. For this case, the devices are supposed to operate in the band [1500, 2500] MHz. As regards the discretization of the linear integral equation in Equation (1) and the evaluation of the matrix associated with the continuous operator A, the Method of Moments with pulses basis functions and point matching in the data space has been applied (e.g., see [34]).

### 4.1. Resolution Results

In this section, we examine the imaging performance that can be achieved when solving the inverse source problem with the sensing configurations A, B and C. The analysis is performed in terms of spectral content of the relevant radiation operator. Of course, a similar study cannot be accomplished for the image-fusion strategies (D and E) owing to the intrinsic non-linearity introduced by the image-fusion process. A comprehensive comparison between all data processing strategies will be carried out in Section 4.2 on the basis of full-wave synthetic data. As for the computation of the radiation operators, the narrow band [2400, 2483.5] MHz is discretized into 10 uniformly spaced samples while the wide band [1500, 2500] MHz is sampled into 50 uniformly spaced frequencies.

**Figure 4:**
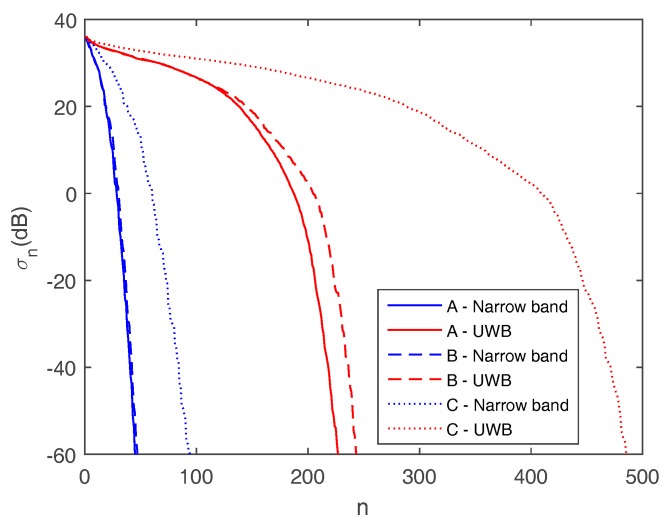
Singular values (in decibels) of the radiation operator for the sensing strategies A, B, C in the narrow band and Ultra Wide Band (UWB) cases.

The curves plotted in Figure 4 display the singular values of the radiation operator for sensing strategies A, B, C in both narrow band and UWB cases. It is observed that all curves exhibit an abrupt decay beyond a critical index. The faster decay of singular values takes place for the configuration A in the narrow band case (solid blue curve). This means that the achievable information that can be extracted from the data is reduced with a severe limitation on the spatial resolution. The sensing configuration B in the narrow band case (dashed blue curve) is characterized by singular values that are very similar to those achieved for strategy A and a narrow band source. As a result, tilting the array with respect to the horizontal direction is not expected to produce a relevant resolution enhancement. When considering the two arrays strategy C in the narrow band case (dotted blue curve), it emerges that the decay of the singular values is slower compared to the cases A and B. Indeed, as a result of the spatial diversity provided by array data fusion, a clear increase in the number of singular values arises for a fixed threshold on the amplitude of the singular values. Therefore, a resolution enhancement is expected despite the limited bandwidth available. The red curves refer to the UWB sources. In particular, the red solid line refers to the horizontal array configuration (A), the red dashed curve refers to the tilted-array configuration (B), and the red dotted curve is concerned with the array data fusion (C). As a general consideration, the possibility of collecting data in a wide frequency range increases considerably the number of significant singular values. Consequently, a notable resolution enhancement is expected in comparison to the narrow band sensing strategies. Moreover, as already found in the narrow band case, the two arrays strategy (C) is characterized by a much higher number of singular values compared to the single-array configurations A and B. These latter configurations perform quite similarly also in the UWB case.

**Figure 5:**
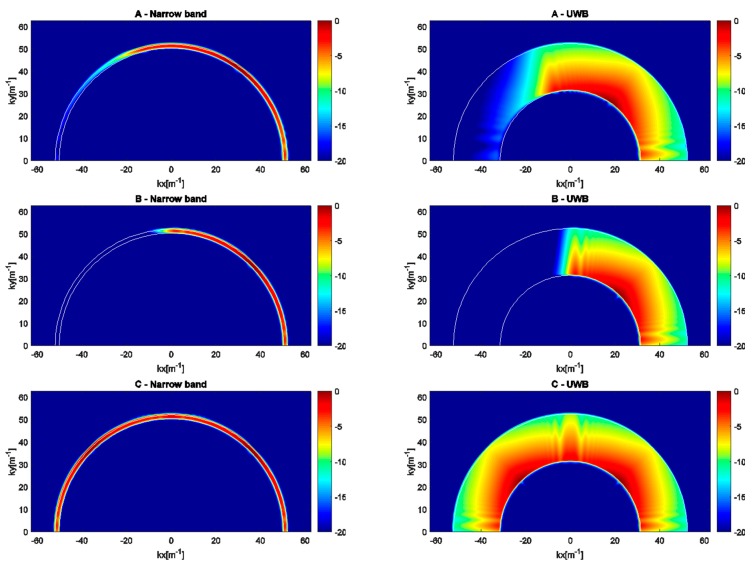
Normalized spectral content (in decibels) achievable with the sensing strategies A, B, C in the narrow band (**left panels**) and UWB (**right panels**) cases.

The images plotted in Figure 5 display the normalized amplitude of the spectral content evaluated via Equation (12). In each figure, the superimposed lines delimit the theoretical spectral set, i.e., the set of spatial frequencies of the unknown current distribution that can be reconstructed from the data. Under a far-field approximation and the assumption of an infinitely long array, such a set is the annular ring delimited by the circumferences with radii $k_{0,min}$ and $k_{0,max}$, with $k_{0,min}$ and $k_{0,max}$ being the propagation constants in free space at the minimum and maximum frequencies, respectively [44].

As a general consideration, the radiation operator acts always as a low-pass filter along *x* and as a band-pass filter along *y*. Therefore, only a smoothed version of the unknown current distribution can be retrieved. Most notably, the spatial filtering is more severe for narrow band sources (left panels of Figure 5) despite an improvement in the spectral coverage being provided by array data fusion (C) compared to single-array configurations (A and B), thanks to the increased spatial diversity. It must likewise be stressed that the actual spectral content in single-array configurations (A and B) is only a subset of the theoretical spectral set (annular ring) because of the limited aspect angle under which the investigation region *D* is probed.

As for the UWB cases (right panels of Figure 5), the spectral coverage is considerably enlarged since the wide frequency bandwidth allows recovery of some low-frequency spatial harmonics of the source current distribution. Therefore, the spatial resolution is greatly improved.

Based on the previous results, it is understood that imaging a narrow band source is more challenging compared to a UWB source. Indeed, the poor resolution deriving from the reduced spectral coverage (limited bandwidth and aspect angle) is expected to affect the localization performance adversely. This motivates the need for array data/image-fusion strategies capable of increasing the information content of data and images to improve the system’s performance.

### 4.2. Localization Results

In this sub-section, we report the imaging and localization results achieved for the indoor scenario and measurement arrangements previously shown in Figure 3. The numerical examples are based on full-wave synthetic data generated by using the numerical solver GPRmax2D [45]. For each source position and sensing configuration in Figure 3, the total field data collected by the antenna arrays are transformed in the frequency domain over the considered frequency bands and corrupted by Additive White Gaussian Noise. The signal-to-noise ratio on the data has been fixed at 10 dB. The data inversion is carried out according to the adjoint inversion scheme (see Equation (3)), but only the tomographic images related to dataset 1, 6, 11, 16, 21, 26 and corresponding to different source positions are shown for the sake of brevity. These points are chosen uniformly spaced along the target’s trajectory. Nevertheless, as will be shown, the performance of the proposed localization strategies will be tested for all the considered source positions.

**Figure 6:**
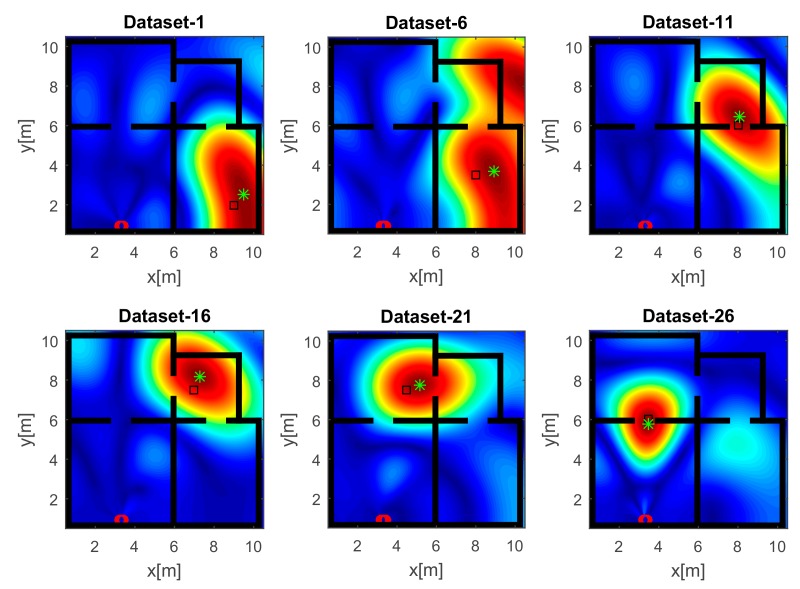
Tomographic images of a narrow band source achieved with the horizontal array (A). Array (red circles); true source position (black square); estimated source position (green asterisk). Color scale is [0, 1].

The images displayed in Figure 6 have been obtained with the horizontal array (A) and a narrow band source. In agreement with the analysis accomplished in Section 4.1, the reconstructions suffer from a poor resolution. Despite that, a large spot appears close to the true source position (black square), so detecting the spot peak allows estimating the source position (green asterisk). It must be pointed out that when the source is far from the receiving array along *x*, the resolution is very low and the reconstruction exhibits a very large spot whose extent is about 10 m along *y* (top middle panel of Figure 6). This result is a consequence of the narrow frequency band and the limited aspect angle and it may lead to localization inaccuracies. When the source and the receiving arrays are in the LOS condition (bottom right panel of Figure 6), the resolution is better and the estimated position is very close to the true value; conversely, a higher positioning error is observed in the NLOS condition (top middle panel of Figure 6). This behavior is expected since, in the NLOS condition, the RF signal undergoes transmission through walls and multipath phenomena that are not taken into account in the inverse source model.

**Figure 7:**
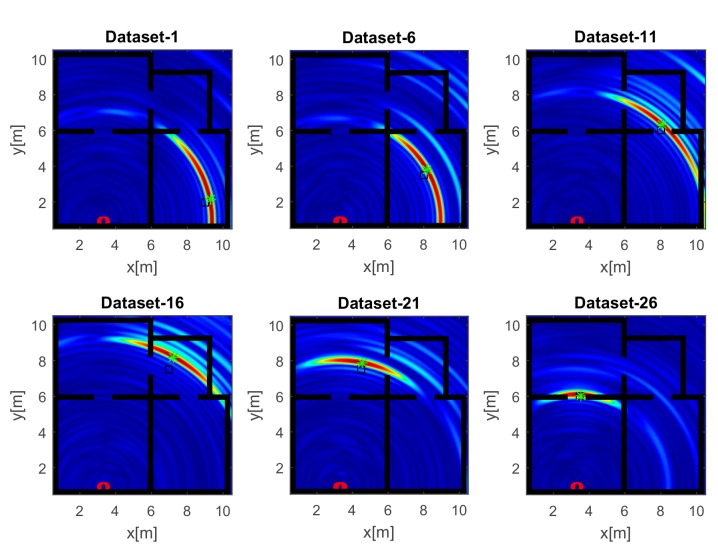
Tomographic images of a UWB source achieved with the horizontal array (A). Array (red circles); true source position (black square); estimated source position (green asterisk). Color scale is [0, 1].

The reconstructions in Figure 7 refer to the strategy A and a UWB source. Unlike the images in Figure 6, the wide bandwidth provides a clear benefit in terms of smaller spot size along the range. However, owing to the limited aspect angle, the source reconstruction resembles an arc of circumference. Furthermore, the side lobes in the images have a lower intensity even when the source is far away from the array along the *x* direction (top panels of Figure 7).

**Figure 8:**
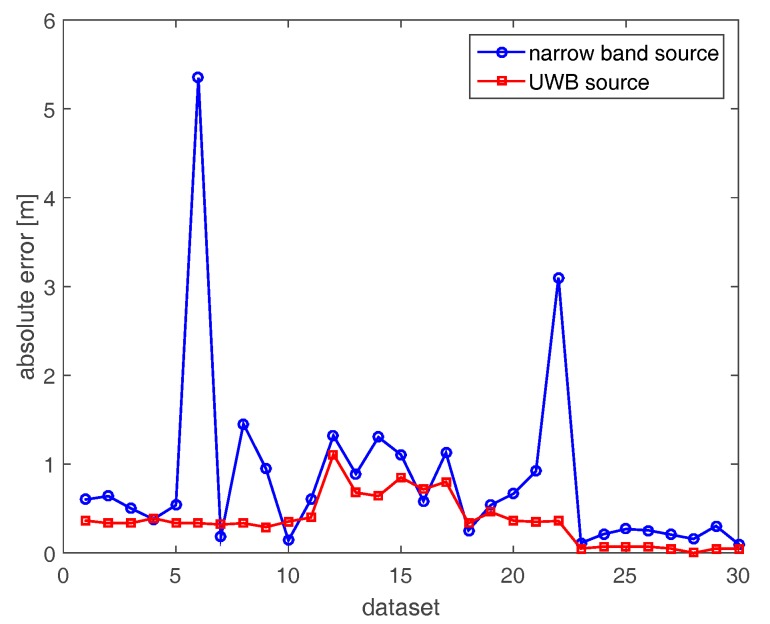
Positioning errors achieved with horizontal array (A).

Figure 8 compares the localization errors attained with the horizontal array in both narrow band and UWB cases. For two datasets (6 and 22), high localization errors (5.5 m and 3 m) arise in the narrow band case because of the very low resolution of the corresponding images. Apart from these two cases, the positioning error is lower than 1.26 m. On the other hand, localization errors in the UWB case (red curve) are generally smaller than their counterparts in the narrow band case.

**Figure 9:**
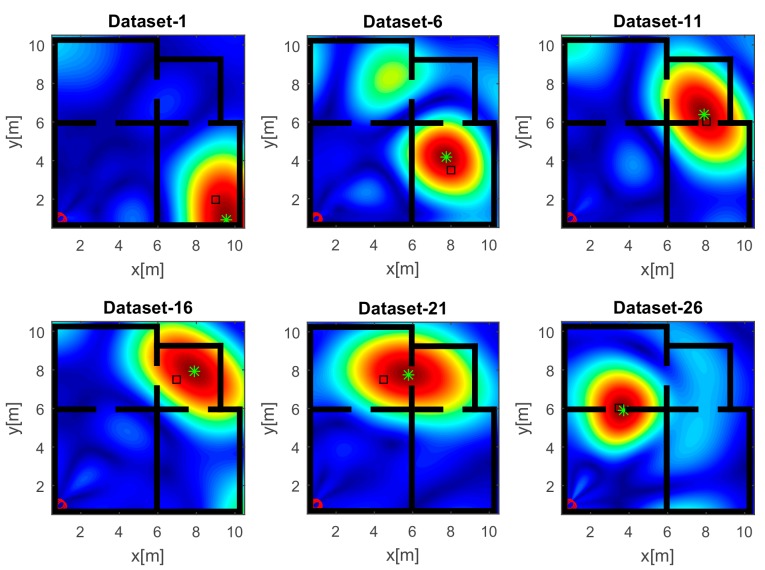
Tomographic images of a narrow band source achieved with the tilted array (B). Array (red circles); true source position (black square); estimated source position (green asterisk). Color scale is [0, 1].

**Figure 10:**
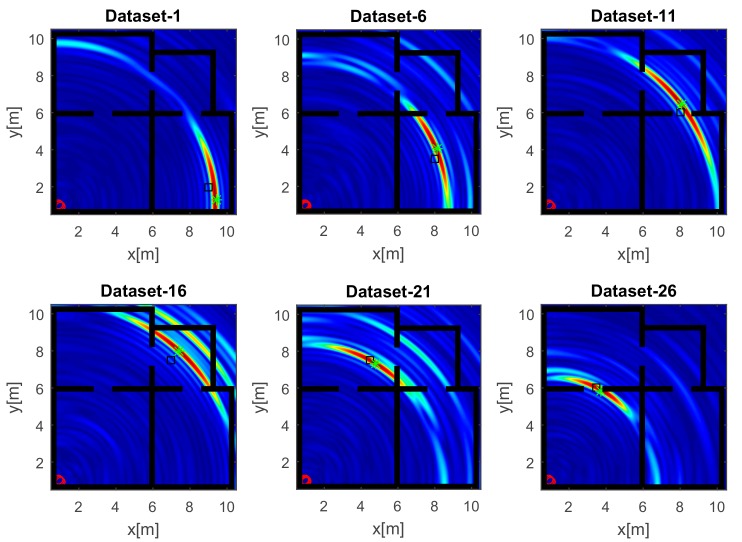
Tomographic images of a UWB source achieved with the tilted array (B). Array (red circles); true source position (black square); estimated source position (green asterisk). Color scale is [0, 1].

**Figure 11:**
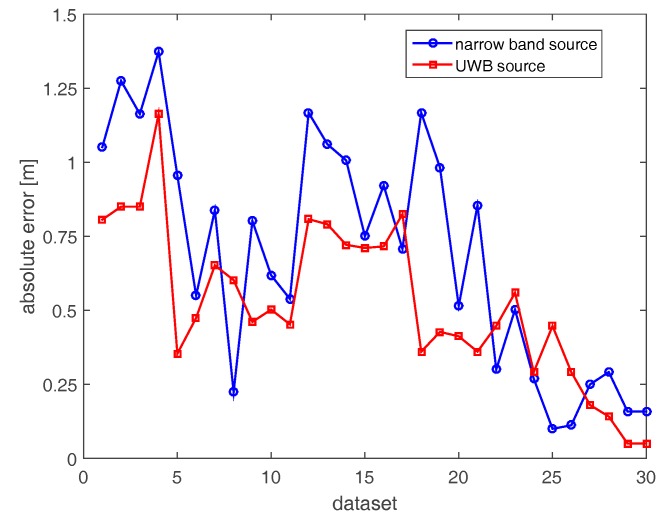
Positioning errors achieved with the tilted array (B).

Figure 9 and Figure 10 deal with the tomographic images attained with the tilted array (B) for the narrow band and UBW source, respectively. The main effect of rotating the array through 145° is to provide a spot size that is more uniform as the source moves along its path (see Figure 9). From an intuitive perspective, tilting the array and placing it in the corner of the room allows for probing of each point of the scene in a similar way. Consequently, the localization becomes more reliable when the source is far away from the measurement array along the horizontal direction. In the UWB case (Figure 10), better resolved source reconstructions are visibly attained for the horizontal array. The comparison of localization errors is reported in Figure 11. The graph confirms that narrow band localization performance with a tilted array is on average still worse than that achieved in the UWB case. However, the differences are less pronounced compared to the positioning accuracy found for the horizontal array (see Figure 8). 

**Figure 12:**
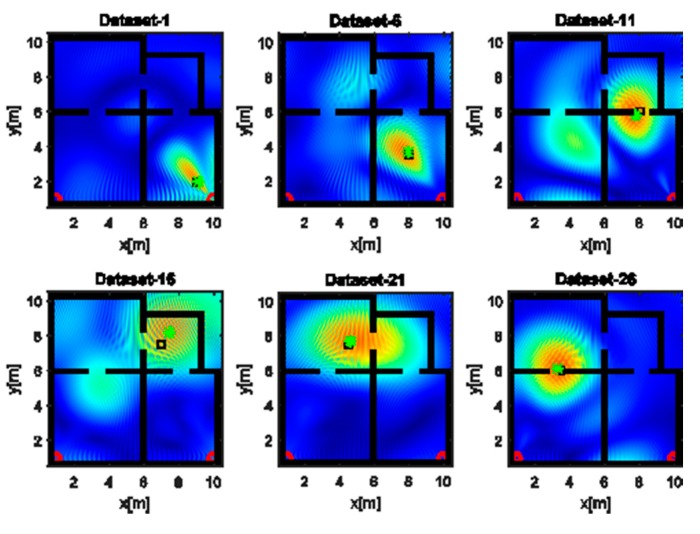
Tomographic images of a narrow band source achieved with array data fusion (C). Arrays (red circles); true source position (black square); estimated source position (green asterisk). Color scale is [0, 1].

**Figure 13:**
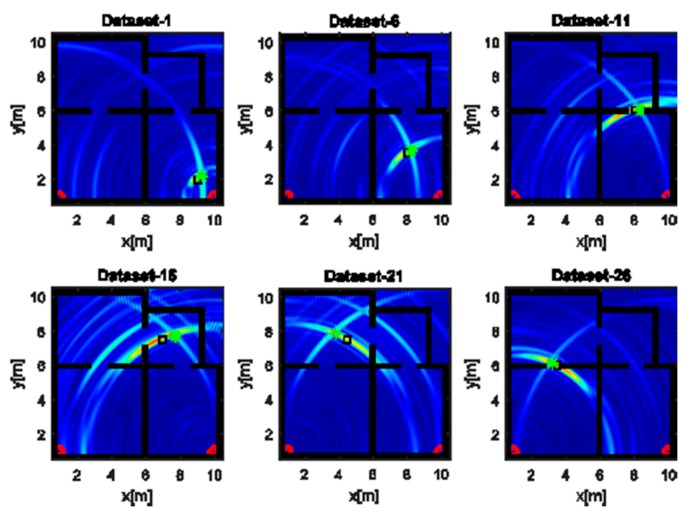
Tomographic images of a UWB source achieved with array data fusion (C). Array (red circles); true source position (black square); estimated source position (green asterisk). Color scale is [0, 1].

**Figure 14:**
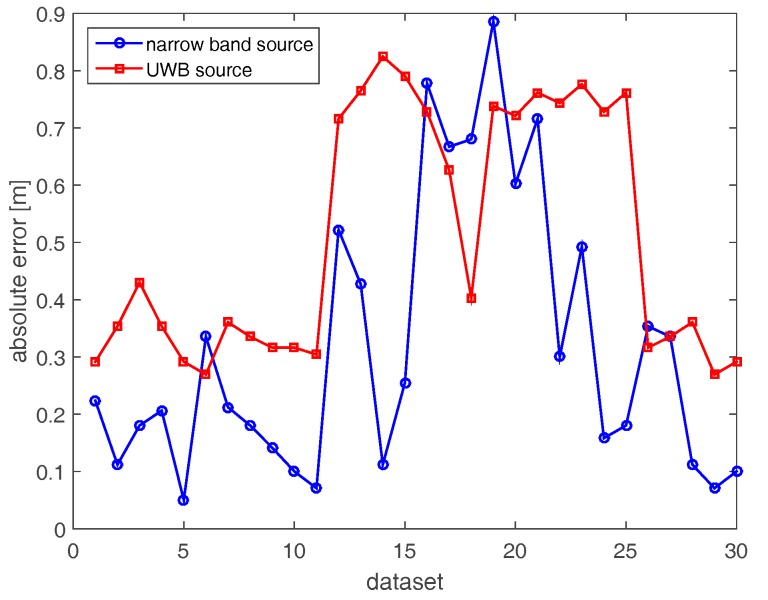
Positioning errors achieved with array data fusion (C).

Here, we examine the localization performance realizable when fusing the data collected by two tilted arrays located at the corners of different rooms (C). Figure 12 displays the images of the narrow band source. In agreement with the spreading of the spectral content (top left panel in Figure 5), the resolution slightly improves compared with the single-array configurations (Figure 6 and Figure 9). Furthermore, the inspected area is probed under dissimilar directions yielding reconstructions with their maxima closer to the true source positions. The tomographic images for the UWB case reported in Figure 13 are better resolved and highlight that the source reconstruction is given by the intersection of the arcs of circumference achieved with each array. Moreover, beside the main spots related to the actual source, additional spots originated by multipath are also present in the images. The comparison of localization accuracies provided by array data fusion is presented in Figure 14. It is notable that the positioning errors are generally lower than those found with single-array configurations A and B (Figure 8 and Figure 11). Remarkably, unlike the previous cases, the narrow band localization error is often lower than its UWB counterpart. This result seems quite counter-intuitive at first sight; however, it can be explained by considering that source reconstructions are given by the intersection of the spots related to each array. Since these spots are well resolved, they are more sensitive to delocalization produced by the uncompensated propagation of the RF signal through building walls.

**Figure 15:**
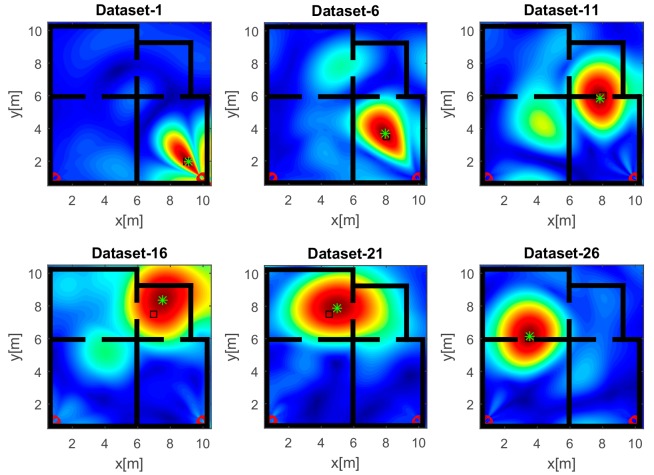
Tomographic images of a narrow band source achieved with additive-array image fusion (D). Arrays (red circles); true source position (black square); estimated source position (green asterisk). Color scale is [0, 1].

**Figure 16:**
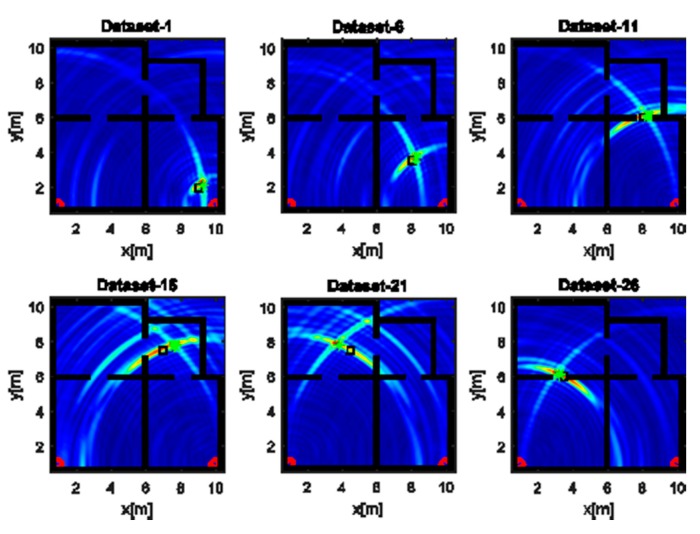
Tomographic images of a UWB source achieved with additive-array image fusion (D). Arrays (red circles); true source position (black square); estimated source position (green asterisk). Color scale is [0, 1].

**Figure 17:**
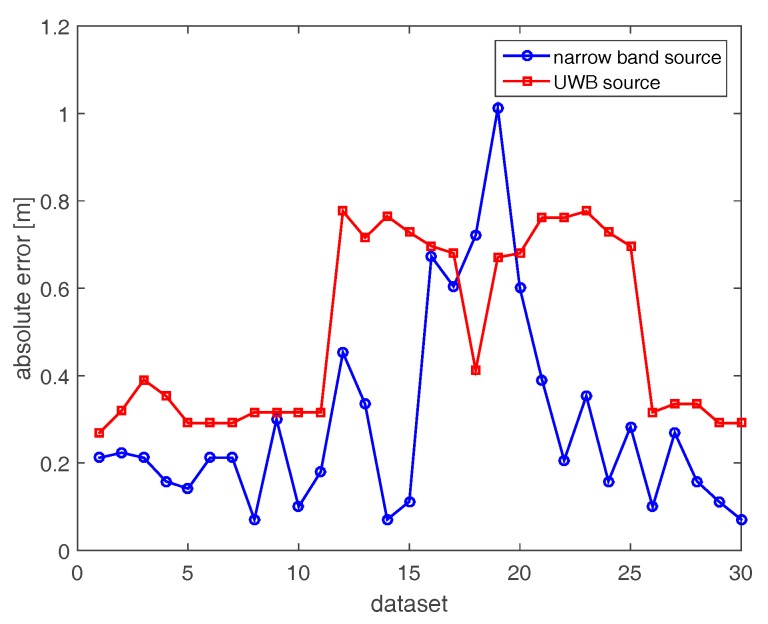
Positioning errors achieved with additive-array image fusion (D).

The results reported in Figure 15, Figure 16 and Figure 17 regard the localization results provided by the additive fusion of the images achieved separately with two tilted arrays (D). It can be seen that additive fusion in the image domain provides tomographic images (Figure 15 and Figure 16) that are very similar to those found with array data fusion (Figure 12 and Figure 13). Therefore, also for this data-processing strategy narrow band localization appears to be more robust with respect to uncompensated wall propagation and multipath (see Figure 17).

**Figure 18:**
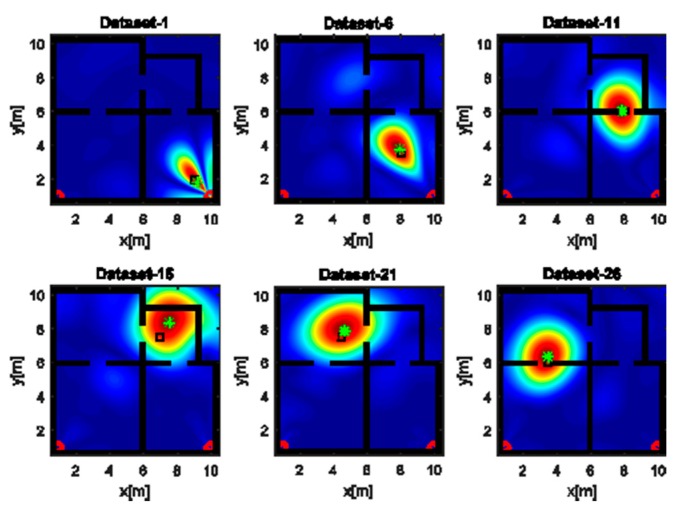
Tomographic images of a narrow band source achieved with multiplicative array image fusion (E). Arrays (red circles); true source position (black square); estimated source position (green asterisk). Color scale is [0, 1].

**Figure 19:**
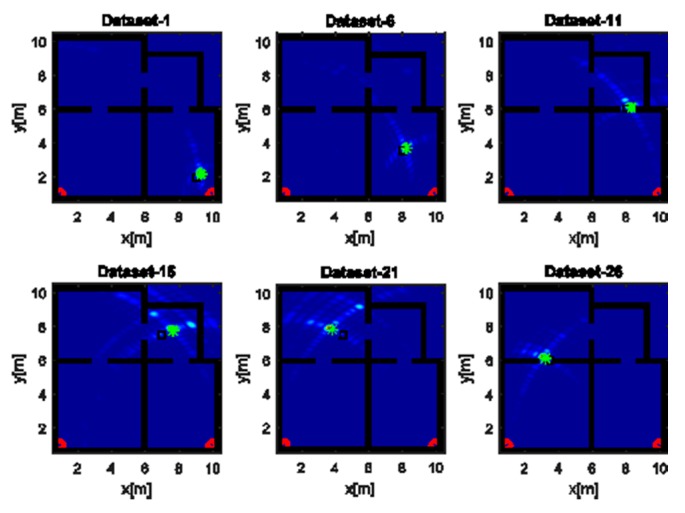
Tomographic images of a UWB source achieved with multiplicative array image fusion (E). Arrays (red circles); true source position (black square); estimated source position (green asterisk). Color scale is [0, 1].

**Figure 20:**
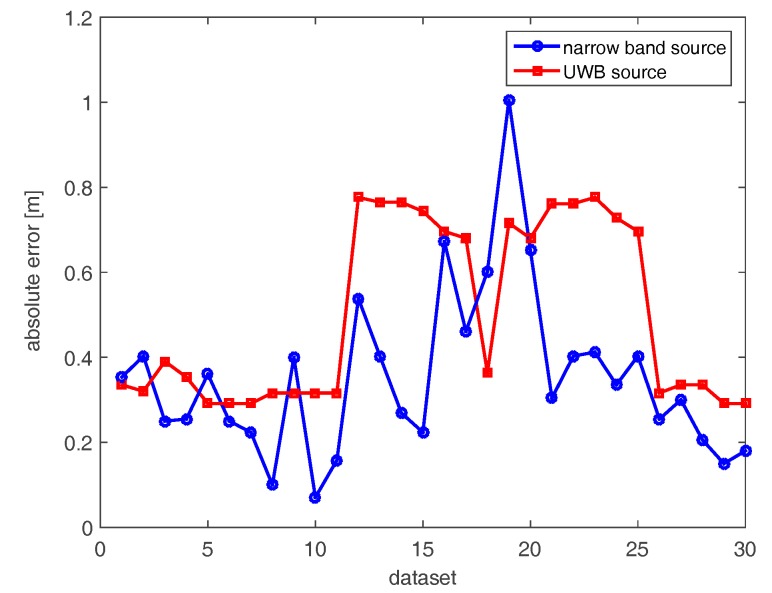
Positioning errors achieved with multiplicative array image fusion (E).

Finally, Figure 18, Figure 19 and Figure 20 are concerned with the multiplicative-array image fusion strategy (E). With regard to the tomographic images, the overall effect of multiplicative fusion is to provide cleaner source reconstructions which are characterized by a smaller spot size and lower amplitude of the side lobes. This is particularly true for the images attained in the UWB case due to the higher resolution of the images involved in the fusion process. As for the localization results in Figure 20, the narrow band case generally provides more reliable results due to its major robustness with respect to delocalization produced by uncompensated propagation through walls.

In conclusion, Table 1 and Table 2 summarize some localization error indicators (mean, maximum and root mean square values) that allow a more quantitative comparison of the performance of each measurement and processing strategy considered so far. It turns out that narrow band source localization with a single array has average accuracy in the order of a meter. Of course, the positioning precision can be increased up to a few tens of centimeters by fusing the data or the images obtained with two receiving arrays properly deployed on the border of the scene under investigation. UWB source positioning accuracy is generally higher than narrow band source localization when deploying a single array. However, multi-array UWB data and image fusion based on a blind inverse source imaging approach does not yield always superior results compared to the narrow band case.

A final consideration deals with the computation complexity related to the proposed data-processing strategies. In particular, the adjoint inversion scheme defined by Equation (3) has been chosen because of its computational efficiency, which renders it a suitable candidate for real-time data processing. For a fixed scenario and sensor array set-up, the matrix associated with the continuous operator is usually pre-computed and stored in a file, so that the solution is readily evaluated as a matrix product. The complexity related to the evaluation of the operator matrix is $O\left(N_{s}N_{f}N_{x}N_{y}\right)$, where $N_{s}$ is the total number of sensors; $N_{f}$ is the number of frequencies; and $N_{x}$ and $N_{y}$ are the numbers of pixels along *x* and *y*, respectively. Therefore, the computation time depends on the size of the image area and the number of data (number sensors times number of frequencies). As regards the numerical experiments described above, a code was developed under the MATLAB environment. The computation times of the operator matrix on a standard laptop equipped with an Intel Core I7 processor and 8-GB RAM are summarized in Table 3 for single-array and two-array configurations as well as in narrow band and UWB cases.

## 5. Conclusions

This work has addressed active radio-frequency source localization in indoor environments. The problem has been handled by means of a blind inverse source imaging approach, which does not require prior knowledge of the scene under investigation. Different measurement configurations and data-processing strategies have been analyzed and compared in terms of spatial resolution and localization accuracy in the case of both narrow band and UWB sources. It has been shown that narrow band source localization with a single measurement array has a typical accuracy around 1 m at 2.4 GHz. However, multi-array data and image fusion offer the possibility of improving the positioning precision up to a few tens of centimeters. The proposed localization strategies are robust with respect to random noise and the main source of error has to be attributed to uncompensated propagation through walls when the source is in the non-line of sight region. Notably, multi-array UWB localization based on a blind inverse source imaging approach does not provide necessarily superior performance compared to the narrow band case. Indeed, it appears more sensitive to the delocalization effect originated by the uncompensated propagation of the wireless signal through building walls.

The enhancement of the proposed localization strategies in the non-line of sight conditions possibly based on a partial knowledge of the scenario and/or soft calibration measurements will be the subject of future research. Furthermore, the proposed algorithms will be compared to existing state-of-the-art approaches at parity of test conditions.

## Figures and Tables

**Table 1:** Localization error indicators in meters: narrow band source.

Configuration	Mean	Maximum	RMS
Horizontal array (A)	0.99	5.50	1.66
Tilted array (B)	0.71	1.42	0.82
Array data fusion (C)	0.29	1.15	0.37
Additive-array image fusion (D)	0.35	1.01	0.36
Multiplicative-array image fusion (E)	0.35	1.00	0.40

**Table 2:** Localization error indicators in meters: UWB source.

Configuration	Mean	Maximum	RMS
Horizontal array (A)	0.37	1.26	0.46
Tilted array (B)	0.52	0.85	0.57
Array data fusion (C)	0.51	0.82	0.55
Additive-array image fusion (D)	0.50	0.78	0.54
Multiplicative-array image fusion (E)	0.50	0.78	0.54

**Table 3:** Computation times of the operator matrix in seconds.

Configuration	Narrow-Band	UWB
Single array	0.5	2.4
Two array	0.9	4.8
